# Sodium Glucose Co-Transporter 2 Inhibitors and the Cardiovascular System: Current Knowledge and Future Expectations

**DOI:** 10.17925/HI.2023.17.2.12

**Published:** 2023-12-01

**Authors:** Ioannis Boutsikos, Eleftherios Beltsios, Bastian Schmack, Ioannis Pantazopoulos, Dimitrios G Chatzis

**Affiliations:** 1. Department of Therapeutics, Alexandra General Hospital, National and Kapodistrian University of Athens, Athens, Greece; 2. Department of Cardiothoracic, Transplant and Vascular Surgery, Hannover Medical School, Hannover, Germany; 3. Department of Emergency Medicine, Medical School, University of Thessaly, Larissa, Greece; 4. School of Medicine, European University of Cyprus, Nicosia, Cyprus

**Keywords:** Diabetic cardiomyopathy, diabetes mellitus, glycosuria, heart insufficiency, sodium glucose co-transporter 2 (SGLT2) inhibitors, renal failure

## Abstract

Diabetic cardiomyopathy is a well-recognized clinical entity and reflects a complex relationship between metabolic substrates and myocardial function. Sodium glucose co-transporter 2 (SGLT2) inhibitors are antidiabetic agents that are found to exert multiple cardioprotective effects. Large clinical trials showed their beneficial effects on patients with heart failure, reducing the rates of rehospitalizations and improving kidney function. The aim of this review is to summarize the latest evidence in the literature regarding the multiple effects of SGLT2 inhibitors on patients across the spectrum of cardiovascular diseases.

Diabetes mellitus (DM) is strongly associated with severe macro-and micro-vascular complications; it is also proven to be a strong independent risk factor for developing heart failure (HF).^1^ In fact, the prevalence of DM is reported to be as high as 20% amongst patients with HF compared with 5–6% in the general population.^2^

Recent studies have shown that DM not only negatively affects patients with ischaemic cardiomyopathy and HF, but also patients with HF with non-ischaemic cardiomyopathy and preserved left ventricular systolic function.^2,3^ The linear relationship of DM and HF was established four decades ago.^4^ Often described as “diabetic cardiomyopathy”, it is mainly characterized by the intensification of atherogenesis, upregulation of the renin-angiotensin-aldosterone system, glycation of interstitial proteins and increased oxidative stress.^56^

Sodium glucose co-transporter 2 (SGLT2) is located in the apical membrane of the proximal convoluted renal tubule and reabsorbs glucose. The overall SGLT2-mediated glucose reabsorption is characterized by high capacity and relatively low affinity of glucose with these transporters.^7^ SGLT2 inhibitors (SGLT2i) are increasingly used as anti-hyperglycaemic agents, either as monotherapy (in patients who are metformin intolerant), or more commonly, as an adjunct therapy to other antidiabetic medications, including insulin.^8^ Their glycosuria effect is irrespective of other epidemiological factors, such as gender, ethnicity, and age.^9^ Moreover, SGLT2i are shown to effectively contribute to weight loss, which is mainly attributed to a decrease in visceral adipose tissue, a property that has recently led to the misuse of the substance by the non-diabetic population.^10^ However, the glucose-l owering effect of SGLT2i are altered mainly by the degree of renal impairment, as they depend on adequate glomerular filtration. The most frequent adverse effects are genital and urinary tract infections due to the extensive glycosuria, while diabetic ketoacidosis is one of the most severe but rare adverse effects.^11^ Apart from their benefit on macro-and micro-vascular complications due to the better glycaemic control, SGLT2i promote osmotic diuresis and natriuresis.^12^ This leads to a decrease in the preload and myocardial strain, which relieves the failing myocardium.^13^

The aim of this article is to summarize the evidence on SGLT2i and major cardiovascular (CV) outcomes across different populations such as patients with heart failure (HF), those with or without diabetes mellitus (DM), individuals with chronic kidney disease (CKD), those with coronary artery disease (CAD), and patients at high cardiovascular risk.

## Mechanism of action

**Figure 1:**
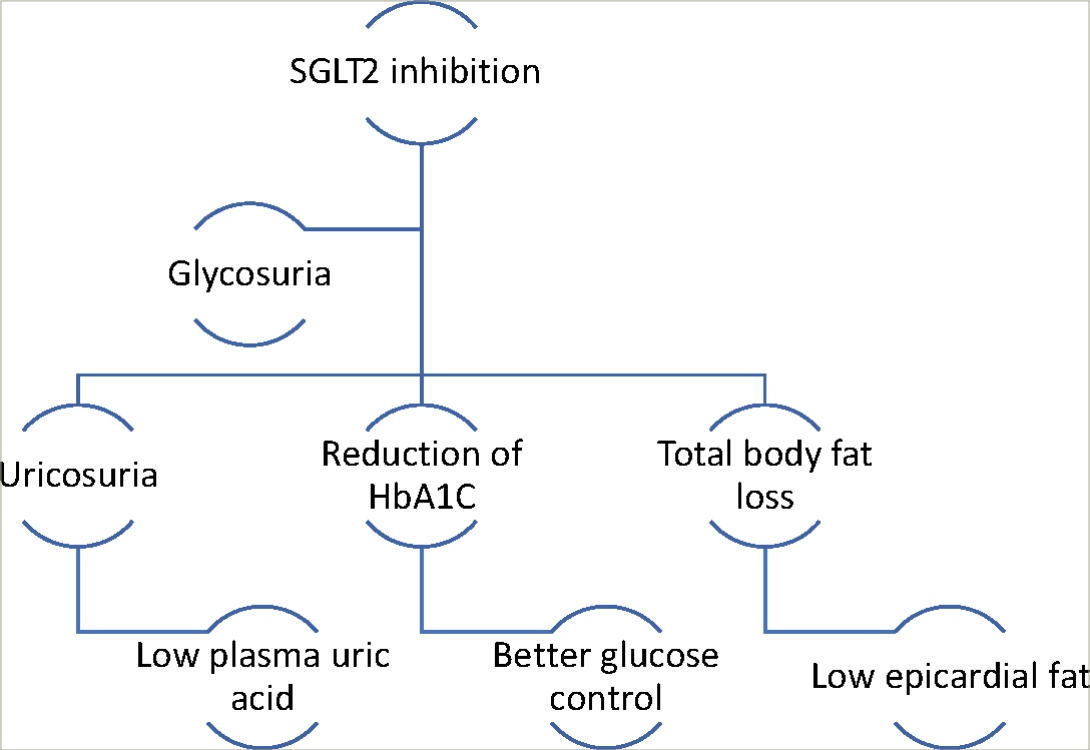
Metabolic effects of SGLT2 inhibition

**Figure 2:**
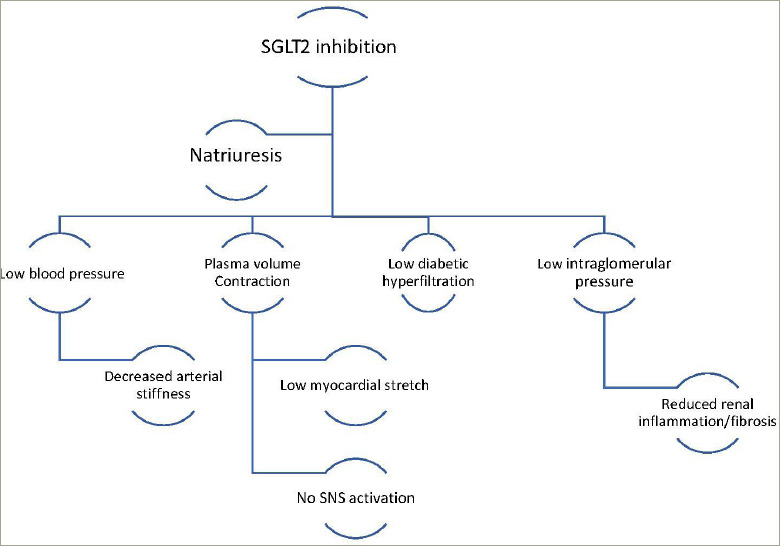
Haemodynamic effects of SGLT2 inhibition

SGLT2 is the main site of glucose reabsorption in the proximal convoluted tubules (>90% glucose reabsorption), but as they are downregulated when serum glucose levels are not high, they do not commonly cause hypoglycaemia and are thus safer compared to other antidiabetic agents such as sulfonylureas.^14^ Furthermore, it has been suggested that excessive glycosuria promotes a 10–15% reduction in serum uric acid, leading to secretion of uric acid in exchange for glucose reabsorption via glucose transporter 9 (GLUT9).^15^ Low serum uric acid is linked to lower CV events, lower rates of renal impairment and decreased blood pressure.^16^ Glycosuria promotes an ongoing catabolic state and adipose tissue loss and consequent reduction of epicardial adipose tissue deposition, which is correlated with atherogenesis.^17^ Notably, the use of empagliflozin and canagliflozin respectively has reduced the microvascular and autonomic complications of diabetes explained by the ameliorated regulation of systemic sympathetic tone.^18,19^ The catabolism of free fatty acids and impaired glucose uptake due to insulin resistance leads to the production of ketones, which could be a significant metabolic substrate for myocardial energy demands (*Figure 1*).^20–22^ SGLT2i increase the concentration of ketone bodies, mostly β-hydroxybutyrate, due to increased glucagon production following glycosuria.^23–25^ Natriuresis plays a significant role in the haemodynamic effects of SGLT2 inhibition. Natriuresis and osmotic diuresis lead to excessive water and sodium excretion, and in this way, the plasma volume contracts, which also causes lower systolic and diastolic blood pressure levels (mean 4 mmHg and 2 mmHg, respectively).^26^ Thus, the detrimental effects of hypertension on vessel walls and on myocardium are diminished.^27^ Low intravascular volume leads to reduced myocardial stretch and workload, allowing the myocardium to function effectively.^28^ Moreover, the intraglomerular pressure is reduced, which reduces renal inflammation and fibrosis, further protecting kidney function^29^ . Diabetic hyperfiltration is a process of diminished distal salt delivery and maladaptive tubuloglomerular feedback, resulting in afferent arteriole dilatation and hyperfiltration.^30^ SGLT2i counteract this process, as sodium is increased in the distal convoluted tubule, and activates the *macula densa*, which is perceived by the juxtaglomerular apparatus as an increase in plasma concentration.^31^ This feedback is triggered by a paracrine mechanism facilitated by adenosine, which contracts the afferent arteriole and blocks hyperfiltration, thus exerting a renal protective role against nephropathy and proteinuria (*Figure 2*).^32,33^ SGLT2i transiently stimulate the renal production of erythropoietin (EPO), which in turn increases the hematocrit.^34^ EPO exerts an established nephroprotective effect via the reduction of oxidative stress and interstitial inflammatory response.^35^

*Low plasma uric acid and epicardial adipose tissue accumulation reduce inflammation and consequent atherogenesis, promoting cardiac protection. Adequate glucose control reduces endothelial dysfunction and microvascular complications*

*HbA1C = hemoglobin A1C; SGLT2 = sodium glucose co-transporter 2.*

The benefit of SGLT2i were initially reported in patients with diabetes from some consecutive landmark trials,^36–39^ which showed the decrease of major adverse CV events (MACE). Following these findings, the use of SGLT2i were then investigated in patients irrespective of their diabetes status, showing again promising results, especially with regards to HF hospitalizations.^36,40,41^ To date, the improvement in clinical outcomes is believed to be due to the pleiotropic effects of SGLT2i on the myocardium and kidneys. A recent study found that SGLT2 is expressed in the cardiomyocytes from patients with end-stage HF irrespective of their HF cause.^42^ Interestingly, SGLT2 is overexpressed in diabetic cardiomyocytes, suggesting that hyperglycaemia may act on cardiomyocytes, increasing SGLT2, and thus supporting the hypothesis of the effect of SGLT2 mediation on metabolic pathways in patients with HF.^43–45^ However, changes in cardiac and renal metabolism and energy use cannot fully explain the benefits of SGLT2i on patients with cardiorenal stress. Molecular and cellular *in vitro* studies suggest that the use of these drugs promote enhanced autophagy, maintain mitochondrial health and mitigate inflammatory and profibrotic pathways.^46–50^

## Patients with heart failure

For the last three years, great emphasis has been put on the pleiotropic effects of SGLT2i on patients with HF with or without type 2 DM. Multiple clinical trials, discussed below extensively (*Table 1*), have proved that SGLT2i are beneficial for the treatment of HF.^36–40,51,52^ Following these results, the European Society of Cardiology added SGLT2i to the treatment algorithm for HF in their latest guidelines.^53^

*Cardiac and renal protection is accomplished via the reduction of excessive volume, which in turn reduces the pressure in the glomerulus and in peripheral vessels. Thus, the workload of the heart and the kidneys is diminished*

*SNS = sympathetic nervous system.*

The first clinical trials included patients with DM and HF with reduced ejection fraction. The Dapagliflozin Effect on Cardiovascular Events– Thrombolysis in Myocardial Infarction 58 (DECLARE–TIMI 58; ClinicalTrials. gov identifier: NCT01730534) trial showed that the patients who received dapagliflozin daily had a lowered risk for HF hospitalization or CV death compared with placebo (HR, 0.83; 95% CI, 0.73–0.95; p=0.005).^40^ Similarly, the Effect of Sotagliflozin on Cardiovascular Events in Patients with Type 2 Diabetes Post Worsening Heart Failure (SOLOIST-WHF; ClinicalTrials. gov identifier: NCT03521934) trial included patients with type 2 DM and worsening HF who received sotagliflozin.^37^ The trial showed that sotagliflozin significantly reduced hospitalizations and urgent visits for HF symptoms.^37^

The following trials included patients with HF who were non-diabetic. Dapagliflozin and Prevention of Adverse Outcomes in Heart Failure (DAPA-HF; ClinicalTrials. gov identifier: NCT03036124) was a remarkable randomized clinical trial evaluating the effect of dapagliflozin versus placebo on patients with HF with reduced ejection fraction and without DM. This trial showed that the patients in the dapagliflozin group had lower rates of HF hospitalizations and urgent visits (HR, 0.74; 95% CI, 0.65–0.85; p<0.001), as well as a lower number of hospitalizations and incidences of CV death (HR, 0.70; 95% CI, 0.59–0.83; 0.82; 95% CI, 0.69– 0.98, respectively).^36^ The DAPA-HF investigators did report that there were specific inclusion and exclusion criteria which could compromise the generalizability of the results. The mean age of included patients was 60–65 years, and thus elderly people were excluded along with patients with acute decompensated heart failure. Sacubitril-valsartan is widely used in HF patients. The percentage of patients who were treated with sacubitril-valsartan was relatively low. This could be a significant confounding factor in the trial’s results as neprilysin inhibition has proven to be one of the cornerstone treatment regimes in HF. Dapagliflozin remains beneficial for patients with HF, even for those treated with sacubitril-valsartan.^54^ However, neprilysin and SGLT2 inhibition represent distinct mechanisms in the pathophysiology pathways of HF.^55^

**Table 1:** Clinical trials with hard cardiovascular endpoints^36–40,51,52^

Clinical trial (NCT identifier)	SGLT2i	Participants (n)	Diabetic status	Heart failure	MACE
DECLARE-TMI (NCT01730534)^40^	Dapagliflozin	17,160	+	↓	↓
DAPA-HF (NCT03036124)^36^	Dapagliflozin	4,744	-	↓	↓
SOLOIST-WHF (NCT03521934)^37^	Sotagliflozin	1,222	+	↓	↓
EMPEROR-Reduced (NCT03057977)^38^	Empagliflozin	3,730	-	↓	NA
EMPULSE (NCT04157751)^51^	Empagliflozin	530	-	NA	↓
DELIVER (NCT03619213)^52^	Dapagliflozin	6,263	-	↓	↓
EMPEROR-Preserved (NCT03057951)^39^	Empagliflozin	5,988	+/-	↓	↓

*All the mentioned clinical trials showed clear benefit compared with placebo regarding the percentage of heart failure hospitalizations and all-cause death.*

*+ = patients with diabetes; - = patients without diabetes;* ↓ = *statistically significant reduction; MACE = major adverse cardiovascular events; n = number of; NA = Data not available; NCT = National Clinical Trials; SGLT2i = sodium glucose co-transporter 2 inhibitors.*

Similarly, Empagliflozin Outcome in Patients With Chronic Heart Failure With Reduced Ejection Fraction (EMPEROR-Reduced; ClinicalTrials. gov identifier: NCT03057977) was a randomized clinical trial with the aim to investigate the effect of empagliflozin compared with placebo on top of standard treatment of non-diabetic patients with HF.^38^ The results showed that the empagliflozin group had lower hospitalization rates in comparison with placebo (HR 0.75; 95% CI, 0.65–0.86; p<0.001) and the total number of hospitalizations were lower for those who received empagliflozin (HR 0.70; 95% CI, 0.58–0.85; p<0.001).^56^ The EMPULSE (Empagliflozin in Patients Hospitalized with Acute Heart Failure Who Have Been Stabilized; ClinicalTrials. gov identifier: NCT04157751) was a randomized clinical trial that evaluated the effect of empagliflozin compared with placebo on patients with acute HF investigating allcause death, HF events and a 5-point difference in the Kansas City Cardiomyopathy Questionnaire (KCCQ). It showed that the initiation of empagliflozin in patients who were hospitalized for acute HF produced clinically beneficial regardless of the degree of symptomatic impairment at baseline and gave improved symptoms and quality of life as early as 15 days after starting therapy and maintaining the effect through 90 days.^51^ However, the 90 day post-discharge follow-up was relatively short, which affects the generalizability of the results. Function classes of patients with HF were assessed using the KCCQ and 6 min walk test.

EMPERIAL-reduced ( ClinicalTrials. gov identifier: NCT03448419) assessed the use of empagliflozin on patients with HF with reduced ejection fraction and 6 min walking distance<350 m.^57^ By the end of the trial, there was no statistical difference in walking distance between the empagliflozin group and placebo group.^58^ Real-world outpatient data suggest that the addition of dapagliflozin to optimal medical therapy of HF with reduced ejection fraction improves advanced echocardiographic parameters including left ventricular global longitudinal strain and myocardial work index.^59^ Two-dimensional speckle-tracking echocardiography provides substantial information concerning the early evaluation of the beneficial effects of SGLT2i on myocardial function.^60^

**Table 2:** Clinical trials with hard renal endpoint^65–68^

Clinical trial (NCT identifier)	SGLT2i inhibitor used	Participants (n)	Diabetic status	CKD	HF hospitalizations	End-stage kidney disease
SCORED (NCT03315143)^65^	sotagliflozin	10,584	+	+	↓	NA
CREDENCE (NCT02065791)^66^	canagliflozin	4,401	+	+	↓	↓
DAPA-CKD (NCT03036150)^67^	dapagliflozin	4,304	+	+	NA	↓
EMPA-KIDNEY (NCT03594110)^68^	empagliflozin	6,609	-	+	NA	↓

*All the mentioned clinical trials showed clear benefit of SGLT2i compared with placebo in patients with CKD.*

*- = patient without condition;* ↓ *= statistically significant reduction; + = patient with condition; CKD = chronic kidney disease; HF = heart failure; SGLT2i = sodium-glucose transporter 2 inhibitors.*

SGLT2i were extensively studied in HF patients with reduced ejection fraction and showed remarkable results.^39,52,61^ However, the efficacy of SGLT2i on HF with preserved ejection fraction remains less certain. The DELIVER (Dapagliflozin in Heart Failure with Mildly Reduced or Preserved Ejection Fraction; ClinicalTrials. gov identifier: NCT03619213) trial was the first clinical trial that investigated the use of dapagliflozin on this group of patients with HF and showed that dapagliflozin reduced the combined risk of worsening HF or CV death (HR 0.82; 95% CI, 0.73–0.92; p<0.001) among them compared with placebo.^52^ In the same manner, EMPEROR-preserved (Empagliflozin outcome trial in patients with chronic heart failure with preserved ejection fraction; ClinicalTrials. gov identifier: NCT03057951) examined the use of empagliflozin on patients with HF with preserved ejection fraction and proposed that empagliflozin reduced the risk of HF outcomes compared to placebo, irrespective of the patients' diabetic status.^39^ According to a meta-analysis of 21,947 patients, SGLT2i should be considered as a foundational therapy in all patients with HF irrespective of ejection fraction or care setting.^61^

## Chronic kidney disease

Patients with HF are at an increased risk of developing chronic kidney disease (CKD).^62^ Those two clinical entities are interdependent, and adverse CV outcomes are of particular importance in patients with CKD as it is the leading cause of death in this clinical population.^63^ This broad impact of CKD involves multiple pathophysiological pathways that link kidney impairment with CV disease and eventually death.

Diabetes and hypertension pose the two main risk factors leading to kidney dysfunction.^64^ The extended research on the use of SGLT2i on patients with HF led to the conduction of clinical trials involving patients with cardiorenal syndrome and reduced estimated glomerular filtration rate (eGFR) (*Table 2*).^65–68^ In the Effect of sotagliflozin on cardiovascular and renal events in participants with type 2 diabetes and moderate renal impairment who are at cardiovascular risk (SCORED; ClinicalTrials. gov identifier: NCT03315143) trial, sotagliflozin was used on patients with HF, type 2 DM and CKD.^65^ The patients in the sotagliflozin group showed less incidence than those on placebo for the composite endpoint of CV death, urgent visits for HF and hospitalizations.^69^

Another important trial was the Evaluation of the effects of canagliflozin on renal and cardiovascular outcomes in participants with diabetic nephropathy (CREDENCE; ClinicalTrials. gov identifier: NCT02065791) trial, which evaluated the effect of canagliflozin on patients with type 2 DM and eGFR >30 to <90 mL/min/1.73 m^2^ and albuminuria.^66^ Surprisingly, canagliflozin lowered the risk for the composite outcome of end-stage kidney disease compared with placebo (HR, 0.70; 95% CI, 0.59–0.82; p<0.0001), and lowered the risk for CV death and HF hospitalizations (HR, 0.69; 95% CI, 0.57–0.83; p=0.0001) as well.^70^

Dapagliflozin was used in a large randomized clinical trial (A study to evaluate the effect of dapagliflozin on renal outcomes and cardiovascular mortality in patients with chronic kidney disease [DAPA-CKD]; ClinicalTrials. gov identifier: NCT03036150) on patients with eGFR = 43 ml/ min/1.73 m^2^, with the aim to investigate the composite of a sustained decline in the eGFR of at least 50%, end stage renal disease or death from renal causes.^67^ Dapagliflozin reduced the occurrence of the composite compared with the placebo (HR, 0.56; 95% CI, 0.45–0.68; p<0.001).^71^

Similarly, in a randomized clinical trial (EMPA-KIDNEY [The study of heart and kidney protection with empagliflozin]; ClinicalTrials. gov identifier: NCT03594110), empagliflozin was used in a broad range of patients with CKD who are were at increased risk of progression to end-stage renal disease.^68^ By the end of the trial, patients in the empagliflozin group had lower risk for kidney disease progression compared with those receiving placebo (HR, 0.72; 95% CI 0.64–0.82; p<0.001).^72^ This was the first trial that included patients with an eGFR of less than 30 ml/min/1.73 m^2^ and an albumin–creatinine ratio (ACR) of <300 mg/g. A large exploratory analysis has shown that empagliflozin slowed the rate of chronic eGFR decline, especially in those patients with severely reduced kidney function.^73^ Dapagliflozin was also used in a large prospective randomized crossover trial (European Union Clinical Trials Register: EU 2017–004641– 25) including patients with CKD and increased albuminuria. The enrolled patients received a combination of dapagliflozin and eplerenone, resulting in a robust and clinically meaningful reduction in albuminuria. Notably, hyperkalaemia was more frequent when eplerenone was used alone compared to the combination of both agents.^74^ SGLT2i and mineralocorticoid receptor antagonists reported an early, acute decline in eGFR >10%, raising safety concerns.^75^ However, this effect resolved after 3 weeks of treatment and was associated with long-term preservation of kidney function.^76^

The results of these trials showed the beneficial effects of SGLT2i on patients with CKD, shedding light upon the complex pathophysiological mechanisms of cardiorenal syndrome and the challenging pharmacotherapeutic approach to this disease.

## Patients with coronary artery disease

Although the use of SGLT2i on patients with HF has been extensively investigated, there is no adequate evidence of the effect of this drug class in patients with CAD or post-acute myocardial infarction (AMI). Data from real-world patients suggested that in the long-term, patients with diabetes and CAD, treated with SGLT2i, had low glucose levels, a high adherence and persistence to treatment and a low number of MACE.^77^ The first trial that assessed the use of empagliflozin after percutaneous coronary intervention following AMI was the Impact of empagliflozin on cardiac function and biomarkers of heart failure in patients with acute myocardial infarction (EMMY; ClinicalTrials. gov identifier: NCT03087773) trial.^78^ It showed that empagliflozin significantly reduced the N-terminal pro-Brain Natriuretic Peptide (NT-proBNP) of patients after AMI, which was also accompanied by a significant improvement in echocardiographic structural and functional parameters.^79^

SGLT2i have been shown to reduce the amount of epicardial adipose tissue, located between the myocardium and the visceral layer of the pericardium, which is known to be metabolically active and related to athersclerosis.^80,81^ Moreover, data from observational studies suggested that the use of SGLT2i is associated with lower incidence of CV events related to in-stent restenosis in patients with diabtes.^82^ A recent study reported that patients with diabetes who received SGLT2i presented lower in-hospital CV death, reduced arrhythmic burden, and lower adverse left ventricular remodeling.^83,84^ Suggested pathophysiological mechanisms include the impact of SGLT2i on excessive inflammatory response, ischaemia/reperfusion injury and generation of reactive oxygen species.^85,86^ In the same manner, patients with diabetes not using SGLT2i were found to over-express specific inflammatory cytokines, such as interleukin-1, interleukin-2 and TNF-a, compared with those patients using SGLT2i. This overexpression reportly lead to a heightened inflammatory response in the blood vessel walls of the non-SGLT2i users.^87,88^ As such, the downregulation of inflammatory burden could potentially lead to atherosclerotic plaque stabilization, reducing MACE.^89^ Further investigation is needed to shed more light upon these cardioprotective properties of SGLT2i on atherosclerosis.

## Patients with high cardiovascular risk

Patients with type 2 DM are at increased risk for CV and renal events. By definition, the glucose-l owering effect of SGLT2i comprise the main mechanism of cardioprotection. However, multiple mechanistic studies on these agents proposed that osmotic diuresis and better mitochondrial function may exert a strong preventive role on diabetic cardiomyopathy.^90^ Nearly, all the hard endpoint clinical trials included patients with established CV disease and the benefit was clear. Primary prevention with SGLT2i is not well studied and thus they are underprescribed.^91^ CANVAS program (CANVAS -canagliflozin cardiovascular assessment study; ClinicalTrials. gov identifier: NCT01032629) was the first clinical trial that involved patients with diabetes with high CV risk and randomly assigned them to canagliflozin or placebo.^92^ The canagliflozin group had a lower risk for the occurrence of CV death, nonfatal myocardial infarction (MI) or nonfatal stroke compared with placebo (HR, 0.86; 95% CI, 0.75‒0.97; p=0.02). Accordingly, the Canagliflozin and cardiovascular and renal events in type 2 diabetes (CANVAS-Renal; ClinicalTrials. gov identifier: NCT01989754) trial showed that the canagliflozin group had lower risk for occurrence of the composite outcome of a sustained 40% reduction in eGFR, the need for renal-replacement therapy or death from renal causes than the placebo group (HR, 0.60; 95% CI, 0.47–0.77).^93^ Although, the clinical effectiveness of SGLT2i in terms of cardiorenal outcomes is proven by multiple clinical trials,^92,93^ they are much more expensive than other antidiabetic agents.^94^ Cost-effectiveness studies suggest that a >70% drop in their cost would support their use as first-line agents in diabetic cardiomyopathy and nephropathy.^95^

## Class effect of SGLT2i

The class effect of a drug is defined by three different concepts – the different substances must share the same chemical structure, similar mechanism of action and identical pharmacological effects. Dapagliflozin, empagliflozin, canagliflozin, ertugliflozin and sotagliflozin share the same structure, inhibit the SGLT2 located on the luminal membrane of proximal tubular cells, and act via glycosuria and subsequent osmotic diuresis.^96^

The established data so far have shown that SGLT2i have a strong class effect on lowering HF hospitalizations and urgent visits. However, the results surrounding MACE remain controversial, as CANVAS, DECLARE TIMI and SCORED, DECLARE and VERTIS CV failed to reduce the risk for MACE.^40,69,93,97,98^

## Ongoing research

Although SGLT2i have shown beneficial effects mostly for patients with HF, independent of their glycaemic status, there are many clinical scenarios that have not yet been addressed. The use of SGLT2i is not fully investigated for patients with type 1 DM (T1DM). One animal study showed that their use in subjects with T1DM was promising for the reduction of CV outcomes, owing to their hypoglycaemic effect.^99^ Moreover, it is of important to mention that even studies that focused on the effect of SGLT2i on kidney function did not include patients that were receiving dialysis or had an eGFR of <20 ml/min/1.73 m.^2^ These extremely frail patients have multiple comorbidities and become easily dysregulated. The cardio-renal-metabolic effect of SGLT2i may benefit such patients by stabilizing their electrolytic disturbances and improving their quality of life.^100^ Following the EMPULSE trial where empagliflozin was studied,^51^ DAPA ACT-HF TIMI 68 ( ClinicalTrials. gov identifier: NCT04363697) is an ongoing clinical trial that evaluates the use of dapagliflozin in patients who have been stabilized during hospitalization for acute heart failure. Sub analysis of the DECLARE TIMI, VERTIS CV, DAPA-HF, and DAPA-CKD trials showed that the group of patients on SGLT2i reported lower incidences of atrial fibrillation and atrial flutter events compared with those in the placebo group, however the clinical significance remains to be evaluated.^101^ The EMPACT-MI ( ClinicalTrials. gov identifier: NCT04509674) is an ongoing double-blind clinical trial that evaluates the effect of empagliflozin in patients with AMI who are at increased risk of developing HF.^102^ As high-risk patients are identified, those who presented with spontaneous MI due to CAD, left ventricular systolic dysfunction (ejection fraction <45%) and signs and symptoms of pulmonary congestion were included in this trial^103^ Patients in the coming years will potentially benefit from the use of these agents , and ongoing research on different pathophysiologic mechanisms might improve their quality of life.

## Conclusion

SGLT2i are pharmacological agents that have been extensively studied in recent years, mostly due to their CV benefits for patients regardless of their glycaemic profile. Their pleiotropic pharmacodynamic properties suggest that they act on a systemic level, affecting multiple organs.^104^ These pharmacotherapeutic regimens have already changed the landscape of chronic conditions such as diabetes, HF and CKD. Their use decreases the rate of HF hospitalizations, reduces MACE and regulates the inflammatory response, leading to atherosclerotic plaque stabilization. SGLT2i improve renal haemodynamics, lower the rate of progression to end-stage kidney disease and control the excessive albuminuria of failing kidneys. In the years to follow, their potential benefit for other clinical entities remains to be answered; ongoing research may shed more light upon different ways SGLT2i may be used to improve patient’s quality of life.
